# MicroRNA-10a is reduced in breast cancer and regulated in part through retinoic acid

**DOI:** 10.1186/s12885-015-1374-y

**Published:** 2015-05-02

**Authors:** Sonja Khan, Deirdre Wall, Catherine Curran, John Newell, Michael J Kerin, Roisin M Dwyer

**Affiliations:** 1 Discipline of Surgery, School of Medicine, Clinical Science Institute, National University of Ireland, Galway, Galway, Ireland; 2 Clinical Research Facility and School of Mathematics, Statistics and Applied Mathematics, National University of Ireland, Galway, Galway, Ireland

**Keywords:** MicroRNA (miRNA), MicroRNA-10a (miR-10a), Breast cancer, Retinoic acid (RA), Retinoic acid receptor beta (RARβ)

## Abstract

**Background:**

MicroRNAs (miRNAs) are short non-coding RNA molecules that play a critical role in mRNA cleavage and translational repression, and are known to be altered in many diseases including breast cancer. MicroRNA-10a (miR-10a) has been shown to be deregulated in various cancer types. The aim of this study was to investigate miR-10a expression in breast cancer and to further delineate the role of retinoids and thyroxine in regulation of miR-10a.

**Methods:**

Following informed patient consent and ethical approval, tissue samples were obtained during surgery. miR-10a was quantified in malignant (n = 103), normal (n = 30) and fibroadenoma (n = 35) tissues by RQ-PCR. Gene expression of Retinoic Acid Receptor beta (RARβ) and Thyroid Hormone receptor alpha (THRα) was also quantified in the same patient samples (n = 168). The in vitro effects of all-trans Retinoic acid (ATRA) and L-Thyroxine (T_4_) both individually and in combination, on miR-10a expression was investigated in breast cancer cell lines, T47D and SK-BR-3.

**Results:**

The level of miR-10a expression was significantly decreased in tissues harvested from breast cancer patients (Mean (SEM) 2.1(0.07)) Log_10_ Relative Quantity (RQ)) compared to both normal (3.0(0.16) Log_10_ RQ, p < 0.001) and benign tissues (2.6(0.17) Log_10_ RQ, p < 0.05). The levels of both RARβ and THRα gene expression were also found to be decreased in breast cancer patients compared to controls (p < 0.001). A significant positive correlation was determined between miR-10a and RARβ (r = 0.31, p < 0.001) and also with THRα (r = 0.32, p < 0.001). In vitro stimulation assays revealed miR-10a expression was increased in both T47D and SK-BR-3 cells following addition of ATRA (2 fold (0.7)). While T_4_ alone did not stimulate miR-10a expression, the combination of T_4_ and ATRA was found to have a positive synergistic effect.

**Conclusion:**

The data presented supports a potential tumour suppressor role for miR-10a in breast cancer, and highlights retinoic acid as a positive regulator of the microRNA.

**Electronic supplementary material:**

The online version of this article (doi:10.1186/s12885-015-1374-y) contains supplementary material, which is available to authorized users.

## Background

MicroRNAs (miRNAs) are an important class of short non-coding RNA molecules proven to have a critical role in mRNA cleavage or decay [1]. miRNAs play crucial roles in a variety of physiological as well as pathological processes including breast cancer [2]. They have been shown to be dysregulated in both tissue and circulation of cancer patients [3-7].

miR-10a is located on chromosome 17q21.32 and is a member of the HOX gene cluster (HOXB and HOXD) [8]. In the miR-10 family, particularly miR-10a/b display relevant roles in developmental pathways which also feature in cancer-related processes [9]. Deregulation of miR-10a has been reported in a number of cancers, including gastric, cervical and thyroid cancer [10-12]. Loss of miR-10a expression was reported in gastric cancer tissues, and in cell lines. This report highlighted a potential tumour suppressor role for miR-10a, partly mediated through DNA methylation [10]. Elevated expression on the other hand was observed in primary cervical tumours. This was associated with an increased risk of developing metastasis facilitated by its binding to phosphatase tensin homologue (PTEN) [11]. Inhibition of both miR-10a and miR-10b was found to promote metastasis in neuroblastoma cell lines [13,14].

In the context of breast cancer, miR-10a expression has been shown to display both oncomiR as well as tumour suppressor roles [15-17]. Elevated miR-10a expression in estrogen receptor (ER)-positive tumours was associated with a longer relapse-free time following Tamoxifen treatment [15]. A study by Chang et al. [16] reported a trend towards increased expression of miR-10a in breast tumours compared to matched tumour associated normal tissues. This study implicated both miR-10a/b expression to be associated with adverse outcomes for breast cancer patients. A study by Pogribny et al. [18] also showed higher expression of miR-10a in breast cancer MCF-7 cell lines with an inbuilt resistance to cisplatin. This analysis identified a potential role for miR-10a in the regulation of cellular proteins, including homeobox family HOXD10, tumour suppressor p27 and ER-alpha (ERα) [18]. Loss of miR-10a expression has been reported by Peres-Riva et al. [17]. A microarray analysis was performed on primary breast tumours from patients with early and late recurrence of the disease. The level of miR-10a was significantly reduced in patients with early breast cancer recurrence, potentially predicting patients at risk of developing recurrence of the disease. A global miRNA profiling study revealed miR-10a to be involved in inhibition of HOXD4 expression in breast cancer cell lines [8].

All-trans-retinoic acid (RA) is a known anti-cancer agent, implicated in a variety of cancers, including lung, head and neck, and haematological malignancies [19-21]. This has been shown through its anti-proliferative, pro-apoptotic and anti-oxidative effects in cell line and animal models [22]. One of the key regulatory targets of RA is retinoic acid receptor beta (RARβ) [23]. It has been revealed to inhibit breast cancer cell proliferation *in vitro* [24,25], and has also been shown to inhibit mammary carcinogenesis in mice [26]. This group and others have reported reduced expression in tumours [27-30]. This protein also has been shown to have a potential tumour suppressor role in breast cancer and is known to dimerize with Thyroid hormone receptor alpha (THRα) [27,31-33].

Studies have reported a link between miR-10a and RA [13,14,34]. In T cells, stimulation with RA alone, or combined with transforming growth factor beta (TGF-β) has been shown to induce miR-10a expression [34]. This type of stimulation by RA has also been reported in a pancreatic and a neuroblastoma cell line model as well as during smooth muscle differentiation [14,35,36]. Based on the conflicting studies to date, the aim of this study was to establish the baseline expression of miR-10a in breast cancer and any potential relationship with RA and L-Thyroxine.

Expression of miR-10a was quantified in tissues from breast cancer patients (*n =* 103), healthy controls (*n =* 30) and patients with benign breast disease (*n =* 35). Any relationship with clinicopathological details was investigated. RARβ and THRα gene expression were previously quantified in the same cohort and any association with miR-10a expression was examined. The impact of RA and L-Thyroxine (T_4_) on miR-10a expression was also determined.

## Methods

### Ethics statement

All experimental procedures involving tissue samples from human participants were approved by the Clinical Research Ethics Committee (University College Hospital, Galway). Written informed consent was obtained from each patient and all clinical investigation was performed according to the principles expressed in the Declaration of Helsinki.

### Clinical samples

Breast tissue specimens (*n =* 168) were obtained at University College Hospital, Galway. The clinical patient samples comprised of 103 malignant tissue biopsies, 30 normal mammary tissue biopsies obtained at reduction mammoplasty, and 35 fibroadenoma tissues which are benign breast disease tissues. Full patient demographics and clinicopathological details were collected and maintained prospectively (Table 1). Samples were immersed in RNAlater® (Qiagen) for 24 hours, then the RNAlater® was removed and the tissue stored at −80°C until required.

**Table 1:** **Patient Clinicopathological details**

Breast Clinicopathological characteristics	Cancer	Fibroadenoma	Normal
Number of patients	103	35	30
Median Patient Age yrs	56 (35–90)	44 (17–62)	46.5 (24–58)
**Menopausal Status**			
Post	72		
Pre	32		
**Histological Subtype**			
Invasive Ductal	78		
Invasive Lobular	11		
*Other*	*14*		
**Intrinsic Subtype**			
Luminal A (ER/PR+, HER2/neu-)	42		
Luminal B (ER/PR+, HER2/neu+)	18		
HER2 Over expressing (ER-, PR-, HER2/neu+)	16		
Triple-Negative (ER-, PR-, HER2/neu-)	16		
*Unknown*	*11*		
**Tumour Grade**			
1	5		
2	32		
3	55		
**Tumour size**			
1	19		
2	39		
3	10		
**UICC Stage**			
Stage 1	23		
Stage 2	36		
Stage 3	21		
Stage 4	10		

### Cell lines and culture conditions

T47D and SK-BR-3 breast cancer cell lines were previously purchased from the American Type Culture Collection (Manassas, VA). T47D cells were cultured in RPMI-1640 media and SK-BR-3 cells were cultured in McCoy’s 5A. Both media types were supplemented with 10% fetal bovine serum (FBS) and 100U/ml penicillin/ 100 μg streptomycin (P/S). Cells were incubated at 37°C and 5% CO_2_ with a media change performed twice weekly and passage every 7 days.

### Total and micro RNA extraction

Breast tissue specimens or cell pellets were homogenised in 1 ml TRIzol® lysis reagent (Invitrogen) as previously described [27]. Total (large and micro) RNA was extracted from malignant (*n =* 103), normal (*n =* 30) and fibroadenoma (*n =* 35) mammary tissue using the RNeasy Mini Kit (QIAGEN) as per manufacturer’s instructions.

### Gene and microRNA analysis

1 μg of large RNA was reverse transcribed using SuperScript III reverse transcriptase enzyme (200U/μl), 0.1 M DTT, RT-5x Buffer, RNaseOut Ribonulease Inhibitor (40U/μl), Random primers (3 μg/μl) and dNTP’s (100 mM)-Promega (Invitrogen, Carlsbad, CA, USA). TaqMan® Gene Expression Assays targeting RARβ and THRα (Table 2) were used in TaqMan® Universal Mastermix (Applied Biosystems). 100 ng of mature miR-10a (Table 2) was reverse transcribed using the MultiScribe™-based High-Capacity cDNA Archive Kit (dNTP 100 mM, RT Buffer 10x, RNase Inhibitor 20U/μl, Stem loop primer 50nM, MultiScribe RT 50U/μl) (Appied Biosystems). The resulting cDNA for both mRNA and microRNA was analysed using the ABI 79000 Fast real-time PCR system (Applied Biosystems). These reactions were carried out in a final volume of 10 μl comprising of 0.7 μl cDNA, 5 μl TaqMan® Universal PCR fast Master Mix (2×), 0.5 μl TaqMan® primer-probe mix (0.2 μM), Forward primer (1.5 μM), and Reverse Primer (0.7 μM) (Applied Biosystems). The RQ-PCR cycle comprised of 10-minute incubation at 95°C followed by a 40 cycles at 95°C for 15 seconds and 60°C for 60 seconds. The use of an Inter-assay control derived from a breast cancer cell line (T47D) on each reaction allowed comparison of data across plates, and all reactions were carried out in triplicate with a standard deviation of < 0.3 between replicates considered acceptable. The relative quantity of mRNA and miRNA expression was calculated using the comparative cycle threshold (ΔΔCt) [37]. The endogenous controls used for gene expression were Mitochondrial Ribosomal Protein L19 (MRPL19) and Peptidyl-Prolyl Isomerase A (PPIA) [38]. For the miRNA analysis, let-7a was employed as an endogenous control [39]. The geometric mean of the Ct value was used to normalise the data and the sample with the lowest expression level was applied as a calibrator.

**Table 2:** **Primer sequence of target mRNAs/miRNAs**

miRNA	Gene locus	Primer sequence
RARß	3p24.2	Forward: CTCCCTCCCTGCCTAACCA
		Reverse: TCCACTGCCTCTTAGCATTTACT
THRα	17q11.2	Forward: TGACCATCGCCGTTAT
		Reverse: GCTTTTGTTGGCGTAC
hsa-miR-10a	17q21.32	Forward: GGAGGGGTACCAGAATCCCATTTTGGCCA
		Reverse: GGAGGAAGCTTGCGGAGTGTTTATGTCAACT

### In vitro stimulation of breast cancer cell lines with all-trans retinoic acid (ATRA) and L-thyroxine (T_4_)

T47D and SK-BR-3 cell lines were seeded at 2.4×10^4^ cells/cm^2^ in a 6-well plate. The following day, the cells were exposed to all-trans Retinoic Acid (ATRA, 0.1 μM, 1 μM, 5 μM) or L-Thyroxine (T_4_, 0.1 μM, 0.5 μM, 5 μM) for 24 hours. This was carried out to establish optimal concentrations for the assay [27]. Once optimal concentrations were established (ATRA, 1 μM, 5 μM and T_4_ 0.5 μM), the assay was then performed in triplicate in both cell lines. The cells were also exposed to a combination of ATRA (1 μM or 5 μM) and T_4_ (0.5 μM) for 24 hours. Controls included cells cultured in the appropriate diluents used for each stimulant. Dimethyl sulphoxide (DMSO, 0.5%) for was used to dilute ATRA. Ammonium hydroxide (NH_4_OH, 0.1%) was the appropriate diluent for T_4_. Cells were harvested at the appropriate time point by trypsination, centrifuged at 120 × g for 4 mins and the cell pellet stored at −80°C. Total large and microRNA was extracted, and the corresponding cDNA analysed using RQ-PCR targeting miR-10a, RARβ and THRα. The endogenous controls used were let-7a for miRNA analysis, and MPRL19 and PPIA for gene expression analysis as previously described. The data was expressed relative to cells cultured in appropriate diluent controls.

### Statistical analysis

All data are presented as Mean (SEM), and graphically represented using box plots and linear scatter plots. A general ANOVA model was used to compare mean responses. Scatter plots were displayed using Linear Regression and Lowess smoother to determine the relationships between different populations. The level of relationship was determined using Pearson correlation coefficients.

## Results

### miR-10a expression in human breast tissues

**Figure 1:**
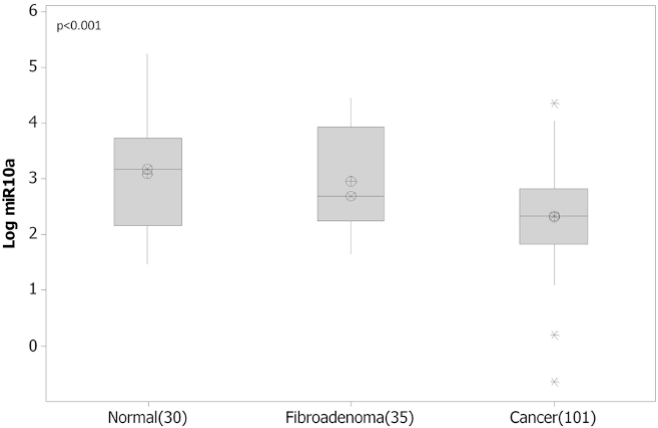
MicroRNA-10a (miR-10a) expression in normal, fibroadenoma and malignant breast tissues. RQ-PCR of miR-10a revealed significantly decreased levels of expression in breast cancer n = 103 (Mean(SEM) 2.3(0.08)Log_10_ Relative Quantity (RQ)) compared to normal tissue n = 30 (3.1(0.17) Log_10_ RQ, p < 0.001) and fibroadenoma tissues (n = 35, 2.9(0.15) Log_10_ RQ, p < 0.001).

MicroRNA extracted from malignant (n = 103), normal (n = 30) and fibroadenoma (n = 35) breast tissue biopsies (Table 1) was analysed by RQ-PCR. Levels of miR-10a were significantly decreased in breast cancer tissues (n = 103, 2.3(0.08) Log_10_ RQ) compared to both normal (n = 30, 3.1(0.17) Log_10_ RQ, p < 0.001) and fibroadenoma tissues (n = 35, 2.9(0.15) Log_10_ RQ, p < 0.001, Figure 1). miR-10a expression was further stratified based on patient clinicopathological details. Expression of miR-10a was not dysregulated across epithelial subtype (p = 0.168), tumour grade (p = 0.299), tumour stage (p = 0.340) or menopausal status (p = 0.126, results not shown).

### RARβ and THRα gene expression in human breast tissues

Expression levels of RARβ and THRα were previously reported by this group, on a total of n = 100 breast tissues, consisting of n = 75 breast cancers, n = 10 fibroadenoma tissues and n = 15 normal breast tissues [27]. Supplementary data shows results from increasing patient sample number to include a total of n = 168 breast tissues for the analysis. This RNA was extracted from an additional n = 27 breast tumours, n = 20 fibroadenoma and n = 15 normal breast tissues was quantified by RQ-PCR targeting RARβ and THRα. RARβ gene expression was found to be significantly down-regulated in breast cancer (n = 101, 0.83 (0.04) Log_10_ RQ) compared to both normal (n = 27, 1.35 (0.09) Log_10_ RQ, p < 0.001) and fibroadenoma tissue (n = 32, 1.49 (0.07) Log_10_ RQ, p < 0.001, Additional file 1: Figure S1.). No significant association was observed with epithelial subtype (p = 0.122), tumour grade (p = 0.363), tumour stage (p = 0.614) or menopausal status (p = 0.635, results not shown).

Levels of THRα were found to be significantly decreased in breast cancer (n = 101, 0.90 (0.03) Log_10_ RQ) compared to both normal (n = 27, 1.50 (0.06) Log_10_ RQ, p < 0.001) and fibroadenoma tissues (n = 32, 1.28(0.07), p < 0.001, Additional file 2: Figure S2.). Further analysis revealed THRα expression was not significantly deregulated across epithelial subtype (p = 0.116), tumour stage (p = 0.859) or menopausal status (p = 0.679, results not shown).

**Figure 2:**
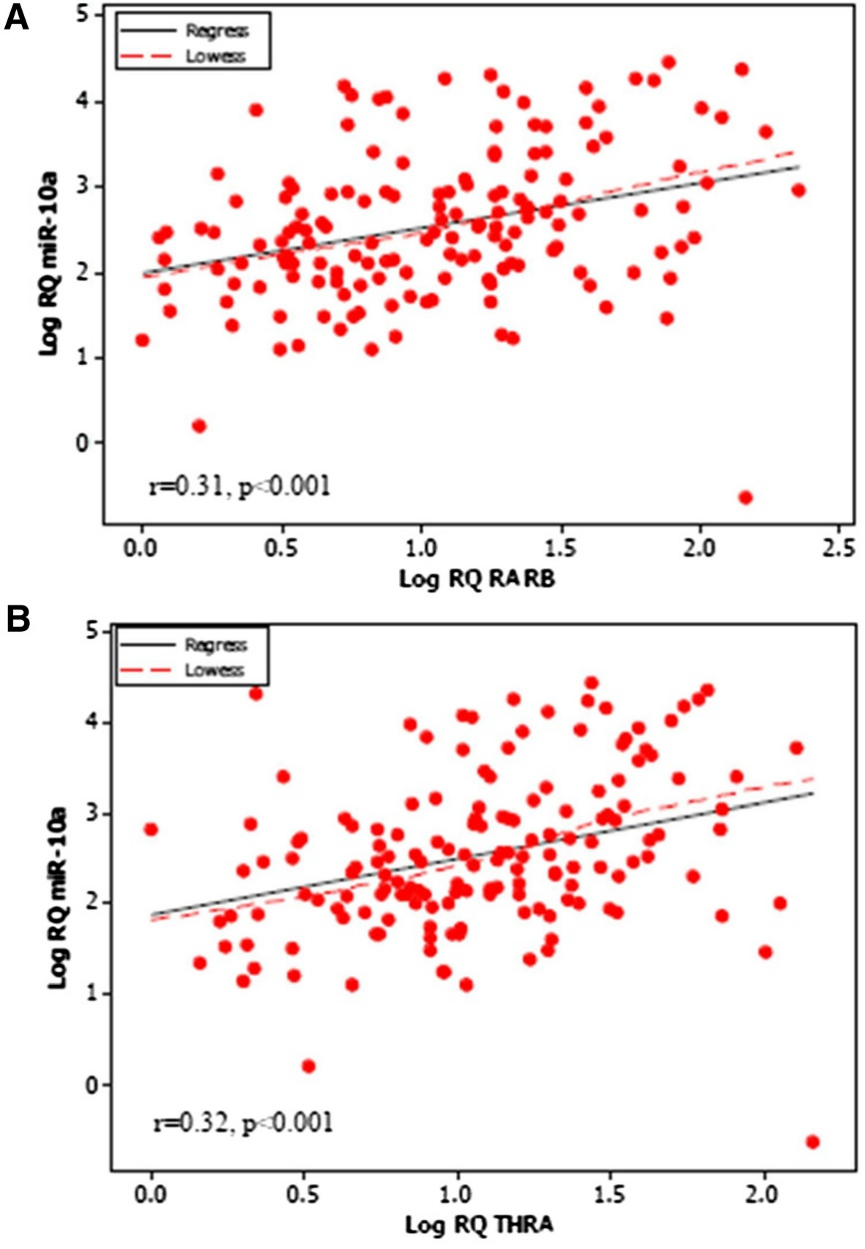
Pearson Correlation of miR-10a and Retinoic Acid Receptor Beta (RARβ) and Thyroid hormone receptor alpha (THRα). **(A)** Pearson correlation of miR-10a and RARβ revealed a significant positive correlation (r = 0.31, p < 0.001). **(B)** The same positive correlation was observed between miR-10a and THRα (r = 0.32, p < 0.001).

Expression of miR-10a across 168 breast tissues was correlated with the gene expression results for RARβ and THRα. A significant positive correlation with RARβ gene expression was observed (r = 0.31, p < 0.001, Figure 2A). miR-10a expression also revealed a robust positive correlation with THRα gene expression (r = 0.32, p < 0.001, Figure 2B).

### In vitro stimulation of breast cancer cells with ATRA or T_4_ alone or in combination

This study was performed to determine the impact of all-trans retinoic acid (RA) and L-Thyroxine (T_4_) on miR-10a expression in vitro. The following concentrations were selected for the analysis, ATRA (1 μM and 5 μM) and T_4_ (0.5 μM). This was determined based on preliminary studies, and reflected the most effective doses. Cells were harvested, and changes in miR-10a expression were quantified by RQ-PCR.

In the case of the T47D cells, miR-10a expression was shown to be stimulated at 1 μM ATRA (2.2 fold, SEM(0.6), p = 0.11) and 5 μM ATRA (2.3 fold, SEM(0.7), p = 0.2). In the presence of 0.5 μM T_4_, no stimulation of miR-10a expression was observed (0.64 fold, (0.08), p < 0.05). When combining both reagents, a significant synergistic increase was shown at 1 μM ATRA+ 0.5 μM T_4_ (3.1 fold, (0.3), p < 0.005) and at 5 μM ATRA+ 0.5 μM T_4_ (3.4 fold, (0.8), p < 0.05).

**Figure 3:**
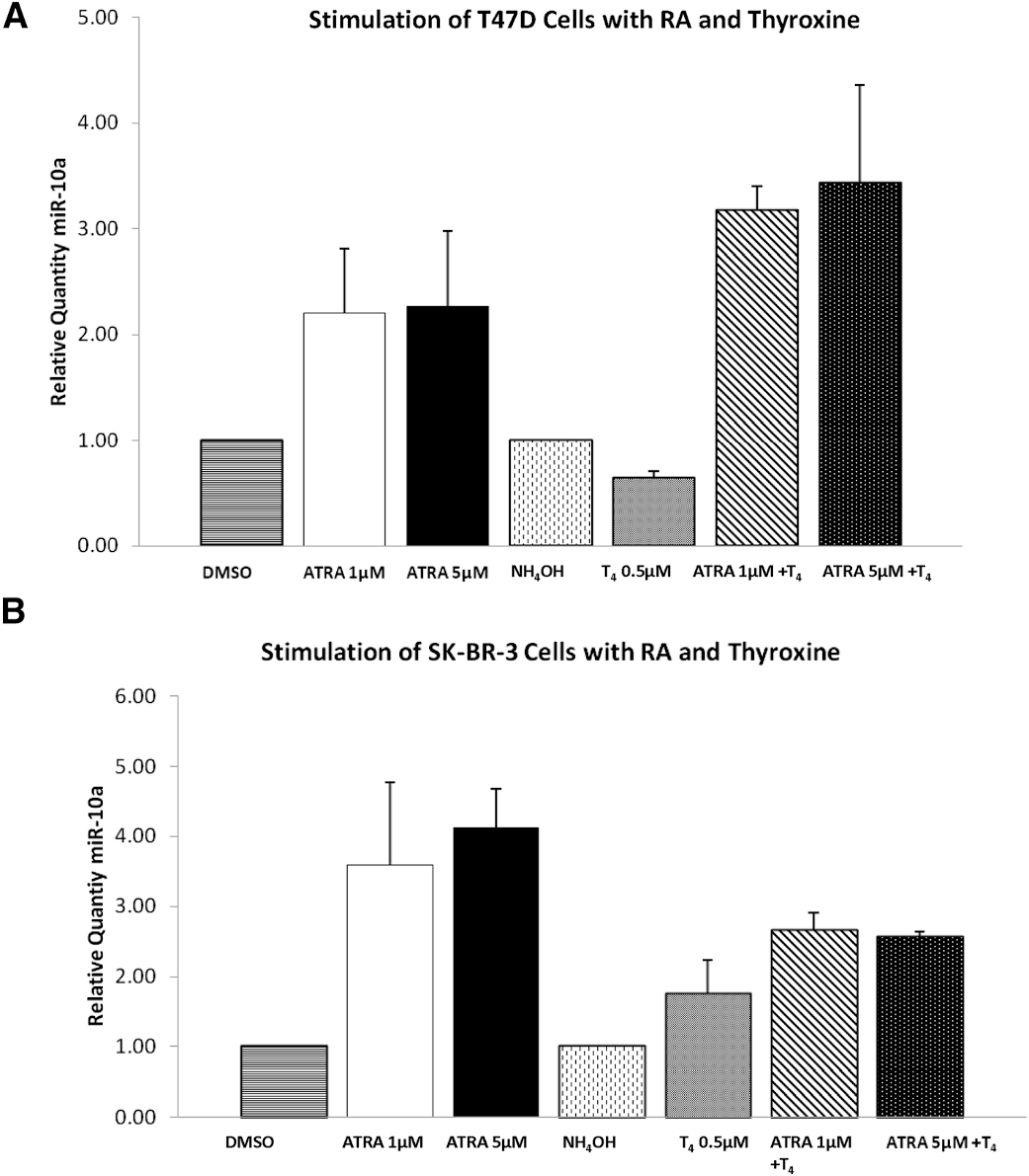
miR-10a Expression in breast cancer cell lines following stimulation with All-trans Retinoic Acid (ATRA) or L-Thyroxine (T_4_) alone or in combination. **(A)** miR-10a expression was quantified by RQ-PCR in T47D cells following 24 hours stimulation with ATRA (1 μM and 5 μM) and T_4_ (0.5 μM) alone or in combination and **(B)** in SK-BR-3 cells.

In the SK-BR-3 cells, ATRA alone had a significant impact on miR-10a expression (3.5-4.1 fold (1.2) p < 0.005, Figure 3B). Similar to the T47D cells, SK-BR-3 cells did not show a change in miR-10a expression following stimulation with T_4_ alone (0.7 fold (0.5)). Combining ATRA and T_4_ resulted in a synergistic impact on miR-10a expression (2.5-2.6 fold (0.2), p < 0.005).

**Figure 4:**
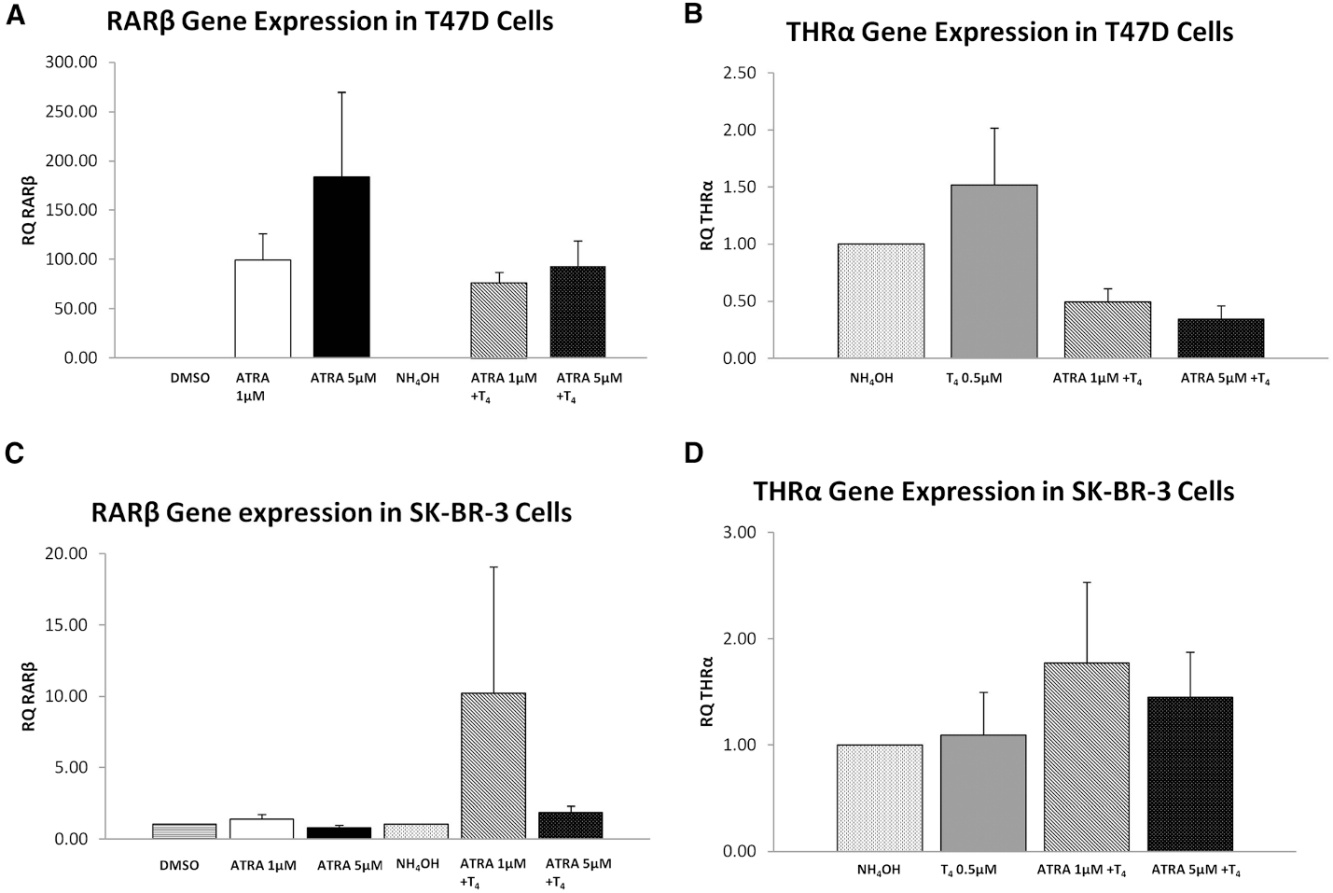
Retinoic acid receptor beta (RARβ) and Thyroid hormone receptor alpha (THRα) gene expression following In Vitro Stimulation in breast cancer cell lines with ATRA or T_4_ alone or in combination. **(A)** RARβ gene expression in T47D cells **(B)** THRα gene expression in T47D cells **(C)** RARβ gene expression in SK-BR-3 cells **(D)** THRα gene expression in SK-BR-3 cells.

The impact of ATRA or T_4_ on receptors was also determined. In the T47D cells, RARβ gene expression was increased by 99–183 fold following stimulation with ATRA alone (Figure 4A). The addition of T_4_ abrogated this effect (76–92 fold increase). Stimulation with T_4_ alone or in combination had no impact on expression of THRα (0.8-1.5 fold, Figure 4B). In the SK-BR-3 cells, combination of ATRA and T_4_ stimulated increased RARβ expression (37–48 fold increase, Figure 4C), with no change observed in THRα (Figure 4D) or RARβ in any other conditions.

## Discussion

Currently there are varied reports on expression of miR-10a in breast cancer. Most recently, a study by Chang et al. [16] quantified miR-10a levels in 108 breast tissues compared to matched tumour associated normal (TAN) tissues, and found no significant changes in malignancy. In contrast, the present study quantified the expression of miR-10a in a total of 168 breast tissues by RQ-PCR. Expression of miR-10a was found to be significantly reduced in breast cancer tissues (n = 103) compared to normal (n = 30) and benign breast disease tissues (n = 35). The different results observed between both studies might be as a consequence of the type of control tissue employed. The present study included control tissues from patients with no history of the disease as well as patients with benign breast disease, while the previous study looked at expression levels in tissue from the tumour-bearing breast of the same patients. The data reported here is also supported by another study, showing reduced miR-10a expression in 71 Formalin fixed, paraffin embedded (FFPE) breast tumour tissues from patients with early recurrence of the disease [17]. This study by Peres-Riva et al. [17] included a microRNA array on FFPE tissues, and determined that loss of miR-10a was associated with the likelihood of developing metastasis. Reduced expression of miR-10a was also previously observed in other types of cancers including gastric cancer and intestinal neoplasia [10,40]. No associations with patient clinicopathological details were observed in the current study.

RARβ is a known tumour suppressor in breast cancer [24,27], confirmed in both cell line and animal models. In the present study, RARβ gene expression was significantly reduced in breast cancer tissues compared to healthy controls. THRα, which is known to dimerize with RARβ [32,33], has also previously been implicated to have a potential tumour suppressor role in breast cancer tissues, confirmed using western blot analysis [27,31]. In the present study, reduced expression was reported in breast cancer tissues compared to healthy controls. A significant positive correlation was observed between miR-10a and RARβ and with THRα in breast tissues. This observation supports a tumour-suppressor role for miR-10a, and instigated further analysis into the potential regulation of miR-10a through RA. Previously miR-10a expression has been shown to be regulated by RA [35], where elevated miR-10a expression in a pancreatic cancer cell line model was reduced using RA inhibitors. In another study, RA elevated miR-10a expression in T helper cells in vitro, resulting in enhanced plasticity of these helper cells [34].

## Conclusions

In the present study miR-10a expression was not affected by stimulation of T47D cells with ATRA or T_4_ alone. Treatment with a combination of ATRA and T_4_ on the other hand showed a greater than 2 fold change in miR-10a expression. In the Her2 amplified SK-BR-3 cell lines however, ATRA alone showed a 2 fold change increase in miR-10a expression compared to the ER positive T47D cells.

Retinoids are used as chemopreventive and anticancer agents because of their ability to regulate cell differentiation, growth and proliferation and apoptosis [22]. This data presented supports a potential tumour suppressor role for miR-10a in breast cancer, and highlights RA alone or in combination with T_4_ as a positive regulator of the miRNA.

## Additional files

Additional file 1: **Retinoic acid receptor beta (RARβ) gene expression across all tissue types.** RARβ is significantly decreased in breast cancer (0.8(0.04) log RQ) compared to both normal (1.3(0.09)log RQ) and benign (1.5(0.07)log RQ) p < 0.001 tissue. Interestingly RARβ is significantly elevated in benign compared to normal and malignant tissue (p < 0.001).
Additional file 2: **Thyroid hormone receptor alpha (THRα) gene expression across all tissue types.** THRα is significantly decreased in breast cancer (0.9(0.03)log RQ) compared to both normal (1.5(0.06) log RQ) and benign (1.3(0.07)log RQ) p < 0.001.

## References

[1] Bartel DP (2004). MicroRNAs: genomics, biogenesis, mechanism, and function. Cell.

[2] Calin GA, Croce CM (2006). MicroRNA signatures in human cancers. Nat Rev Cancer.

[3] Bieche I, Vacher S, Lallemand F, Tozlu-Kara S, Bennani H, Beuzelin M (2011). Expression analysis of mitotic spindle checkpoint genes in breast carcinoma: role of NDC80/HEC1 in early breast tumorigenicity, and a two-gene signature for aneuploidy. Mol Cancer.

[4] Sassen S, Miska EA, Caldas C (2008). MicroRNA: implications for cancer. Virchows Arch.

[5] Kuhling H, Alm P, Olsson H, Ferno M, Baldetorp B, Parwaresch R (2003). Expression of cyclins E, A, and B, and prognosis in lymph node-negative breast cancer. J Pathol.

[6] Rudolph P, Kuhling H, Alm P, Ferno M, Baldetorp B, Olsson H (2003). Differential prognostic impact of the cyclins E and B in premenopausal and postmenopausal women with lymph node-negative breast cancer. Int J Cancer.

[7] Heneghan HM, Miller N, Kelly R, Newell J, Kerin MJ (2010). Systemic miRNA-195 differentiates breast cancer from other malignancies and is a potential biomarker for detecting noninvasive and early stage disease. Oncologist.

[8] Tan Y, Zhang B, Wu T, Skogerbo G, Zhu X, Guo X (2009). Transcriptional inhibiton of Hoxd4 expression by miRNA-10a in human breast cancer cells. BMC Mol Biol.

[9] Lund AH (2010). miR-10 in development and cancer. Cell Death Differ.

[10] Jia HY, Zhang ZY, Zou DL, Wang B, Yan YM, Luo M, et al. MicroRNA-10a is down-regulated by DNA methylation and functions as a tumor suppressor in gastric cancer cells. PloS One. 2014;9(1). doi:ARTN e88057 doi:10.1371/journal.pone.0088057. 10.1371/journal.pone.0088057 PMC3909310 24498243

[11] Zeng TH, Li GL (2014). MicroRNA-10a enhances the metastatic potential of cervical cancer cells by targeting phosphatase and tensin homologue. Mol Med Rep.

[12] Hudson J, Duncavage E, Tamburrino A, Salerno P, Xi L, Raffeld M (2013). Overexpression of miR-10a and miR-375 and downregulation of YAP1 in medullary thyroid carcinoma. Exp Mol Pathol.

[13] Meseguer S, Mudduluru G, Escamilla JM, Allgayer H, Barettino D (2011). MicroRNAs-10a and -10b contribute to retinoic acid-induced differentiation of neuroblastoma cells and target the alternative splicing regulatory factor SFRS1 (SF2/ASF). J Biol Chem.

[14] Foley NH, Bray I, Watters KM, Das S, Bryan K, Bernas T (2011). MicroRNAs 10a and 10b are potent inducers of neuroblastoma cell differentiation through targeting of nuclear receptor corepressor 2. Cell Death Differ.

[15] Hoppe R, Achinger-Kawecka J, Winter S, Fritz P, Lo W-Y, Schroth W, et al. Increased expression of miR-126 and miR-10a predict prolonged relapse-free time of primary oestrogen receptor-positive breast cancer following tamoxifen treatment. Eur J Cancer. 2013;(0). http://dx.doi.org/10.1016/j.ejca.2013.07.145. 10.1016/j.ejca.2013.07.145 23968733

[16] Chang CH, Fan TC, Yu JC, Liao GS, Lin YC, Shih A (2014). The prognostic significance of RUNX2 and miR-10a/10b and their inter-relationship in breast cancer. J Transl Med.

[17] Perez-Rivas LG, Jerez JM, Carmona R, de Luque V, Vicioso L, Claros MG, et al. A microRNA signature associated with early recurrence in breast cancer. PloS One. 2014;9(3). doi:ARTN e91884. doi:10.1371/journal.pone.0091884. 10.1371/journal.pone.0091884 PMC3954835 24632820

[18] Pogribny IP, Filkowski JN, Tryndyak VP, Golubov A, Shpyleva SI, Kovalchuk O (2010). Alterations of microRNAs and their targets are associated with acquired resistance of MCF-7 breast cancer cells to cisplatin. Int J Cancer.

[19] Hong WK, Lippman SM, Itri LM, Karp DD, Lee JS, Byers RM (1990). Prevention of second primary tumors with isotretinoin in squamous-cell carcinoma of the head and neck. N Engl J Med.

[20] Kakizuka A, Miller WH, Umesono K, Warrell RP, Frankel SR, Murty VVVS (1991). Chromosomal translocation T(15–17) in human acute promyelocytic leukemia fuses Rar-Alpha with a novel putative transcription factor, Pml. Cell.

[21] Lippman SM, Benner SE, Hong WK (1994). Retinoid chemoprevention studies in upper aerodigestive tract and lung carcinogenesis. Cancer Res.

[22] Alizadeh F, Bolhassani A, Khavari A, Bathaie SZ, Naji T, Bidgoli SA (2014). Retinoids and their biological effects against cancer. Int Immunopharmacol.

[23] Theodosiou M, Laudet V, Schubert M (2010). From carrot to clinic: an overview of the retinoic acid signaling pathway. Cell Mol Life Sci.

[24] Prakash P, Russell RM, Krinsky NI (2001). In vitro inhibition of proliferation of estrogen-dependent and estrogen-independent human breast cancer cells treated with carotenoids or retinoids. J Nutr.

[25] Rubin M, Fenig E, Rosenauer A, Menendez-Botet C, Achkar C, Bentel JM (1994). 9-Cis retinoic acid inhibits growth of breast cancer cells and down-regulates estrogen receptor RNA and protein. Cancer Res.

[26] Toma S, Isnardi L, Raffo P, Riccardi L, Dastoli G, Apfel C (1998). RARalpha antagonist Ro 41–5253 inhibits proliferation and induces apoptosis in breast-cancer cell lines. Int J Cancer.

[27] Ryan J, Curran CE, Hennessy E, Newell J, Morris JC, Kerin MJ (2011). The sodium iodide symporter (NIS) and potential regulators in normal, benign and malignant human breast tissue. PLoS One.

[28] Widschwendter M, Berger J, Daxenbichler G, Muller-Holzner E, Widschwendter A, Mayr A (1997). Loss of retinoic acid receptor beta expression in breast cancer and morphologically normal adjacent tissue but not in the normal breast tissue distant from the cancer. Cancer Res.

[29] Wu Q, Dawson MI, Zheng Y, Hobbs PD, Agadir A, Jong L (1997). Inhibition of trans-retinoic acid-resistant human breast cancer cell growth by retinoid X receptor-selective retinoids. Mol Cell Biol.

[30] Yang QF, Sakurai T, Kakudo K (2002). Retinoid, retinoic acid receptor beta and breast cancer. Breast Cancer Res Treat.

[31] Silva J, Domínguez G, González-Sancho J, García J, Silva J, García-Andrade C (2002). Expression of thyroid hormone receptor/erbA genes is altered in human breast cancer. Oncogene.

[32] Lee S, Privalsky ML (2005). Heterodimers of retinoic acid receptors and thyroid hormone receptors display unique combinatorial regulatory properties. Mol Endocrinol.

[33] Barton KN, Stricker H, Brown SL, Elshaikh M, Aref I, Lu M (2008). Phase I study of noninvasive imaging of adenovirus-mediated gene expression in the human prostate. Mol Ther.

[34] Takahashi H, Kanno T, Nakayamada S, Hirahara K, Sciume G, Muljo SA (2012). TGF-beta and retinoic acid induce the microRNA miR-10a, which targets Bcl-6 and constrains the plasticity of helper T cells. Nat Immunol.

[35] Weiss FU, Marques IJ, Woltering JM, Vlecken DH, Aghdassi A, Partecke LI (2009). Retinoic acid receptor antagonists inhibit miR-10a expression and block metastatic behavior of pancreatic cancer. Gastroenterology.

[36] Huang H, Xie C, Sun X, Ritchie RP, Zhang J, Chen YE (2010). miR-10a contributes to retinoid acid-induced smooth muscle cell differentiation. J Biol Chem.

[37] Livak KJ, Schmittgen TD (2001). Analysis of relative gene expression data using real-time quantitative PCR and the 2(−Delta Delta C(T)) Method. Methods.

[38] McNeill RE, Miller N, Kerin MJ (2007). Evaluation and validation of candidate endogenous control genes for real-time quantitative PCR studies of breast cancer. BMC Mol Biol.

[39] Davoren PA, McNeill RE, Lowery AJ, Kerin MJ, Miller N (2008). Identification of suitable endogenous control genes for microRNA gene expression analysis in human breast cancer. BMC Mol Biol.

[40] Stadthagen G, Tehler D, Hoyland-Kroghsbo NM, Wen J, Krogh A, Jensen KT (2013). Loss of miR-10a activates lpo and collaborates with activated Wnt signaling in inducing intestinal neoplasia in female mice. PLoS Genet.

