# Supplementary figures and images for: MicroRNA-10a is reduced in breast cancer and regulated in part through retinoic acid
Source: BMC Cancer. 2015 May 2;15:345. doi: 10.1186/s12885-015-1374-y (PMC4425901; doi:10.1186/s12885-015-1374-y)

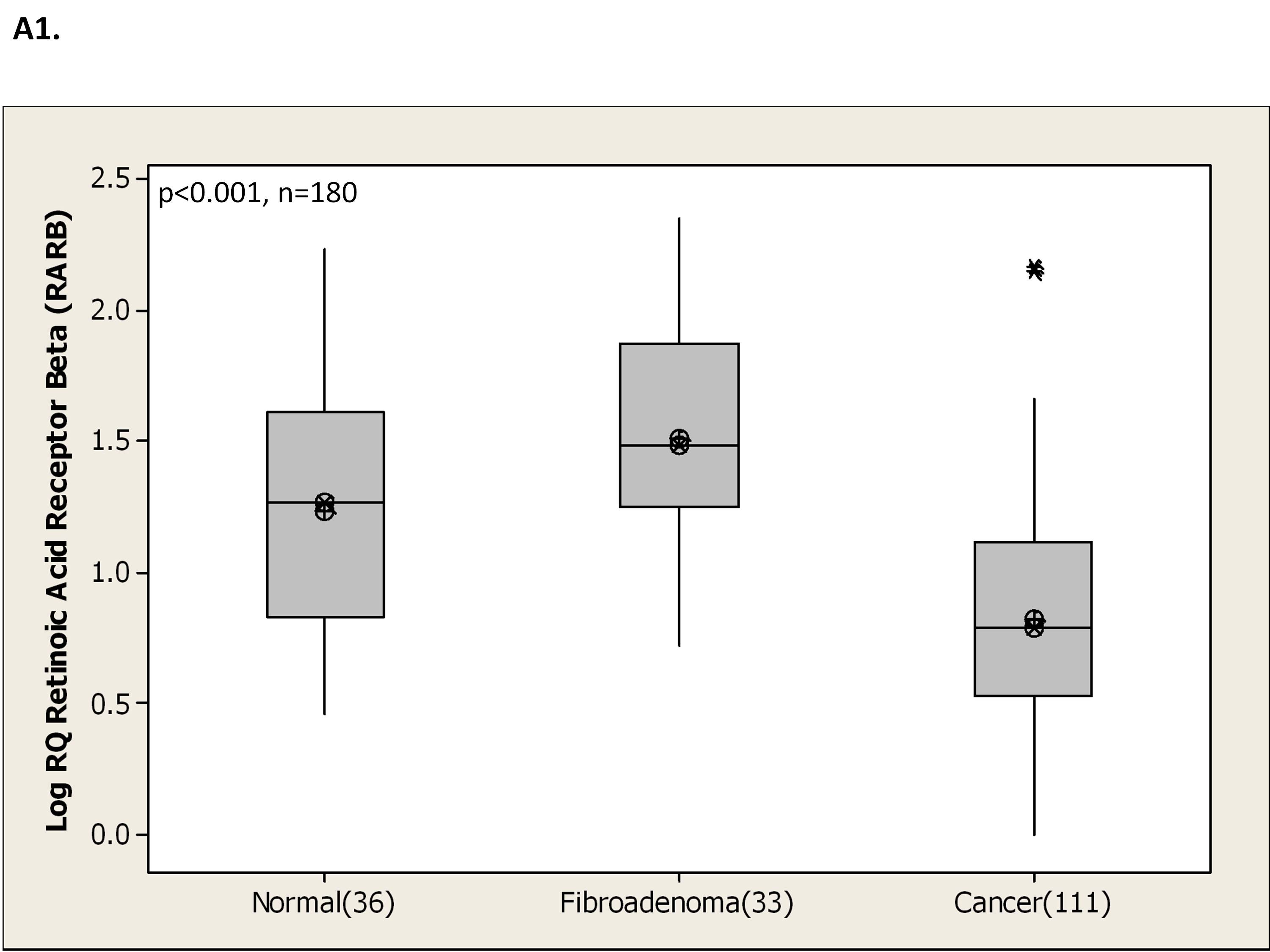

Supplement: Additional file 1: — Retinoic acid receptor beta (RARβ) gene expression across all tissue types. RARβ is significantly decreased in breast cancer (0.8(0.04) log RQ) compared to both normal (1.3(0.09)log RQ) and benign (1.5(0.07)log RQ) p < 0.001 tissue. Interestingly RARβ is significantly elevated in benign compared to normal and malignant tissue (p < 0.001). [file 12885_2015_1374_MOESM1_ESM.jpeg]

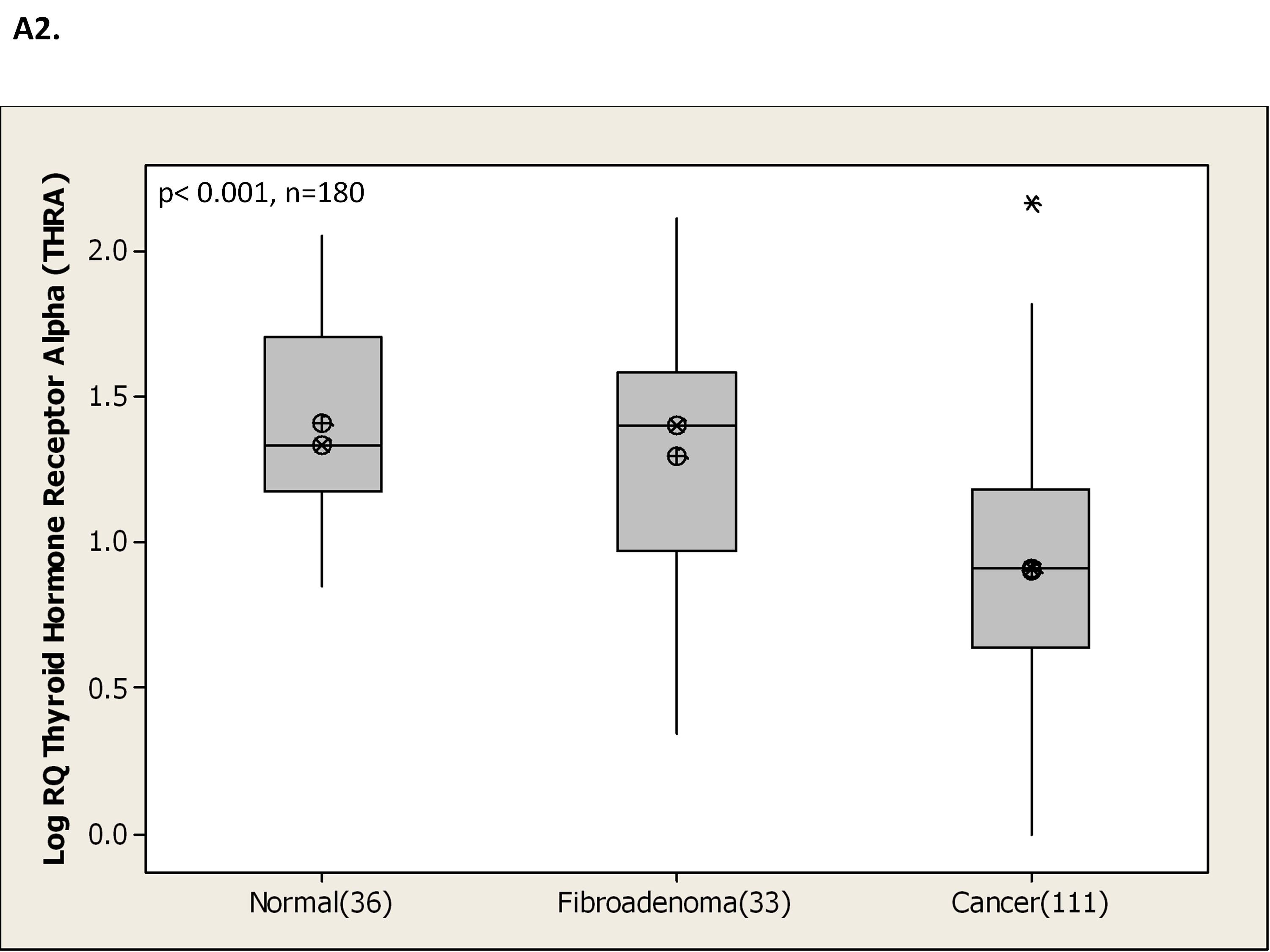

Supplement: Additional file 2: — Thyroid hormone receptor alpha (THRα) gene expression across all tissue types. THRα is significantly decreased in breast cancer (0.9(0.03)log RQ) compared to both normal (1.5(0.06) log RQ) and benign (1.3(0.07)log RQ) p < 0.001. [file 12885_2015_1374_MOESM2_ESM.jpeg]
